# A systematic scoping review of urban food environment research, interventions, and measurement approaches in eight low- and middle-income countries

**DOI:** 10.1186/s12966-026-01884-2

**Published:** 2026-03-05

**Authors:** Amy Margolies, Dorcas Amunga, Esther M. Choo

**Affiliations:** 1 Nutrition, Diets, and Health Unit, International Food Policy Research Institute, Washington, DC USA; 2 International Potato Center, Nairobi, Kenya; 3 Independent Consultant, Seattle, USA

**Keywords:** Urban food environments, Peri-urban food environments, Low- and middle-income countries (LMICs), Nutrition outcomes, Dietary behaviors, Ultra-processed foods

## Abstract

**Background:**

Food environments in low- and middle-income countries (LMICs) are undergoing rapid transformation, particularly in urban and peri-urban settings. These shifts—characterized by changes in food retail landscapes, consumer purchasing behaviors, and the availability and affordability of nutritious foods—have significant implications for nutrition. Yet, the key characteristics of these environments, such as food desirability, convenience, accessibility, and marketing influences, remain underexplored. This systematic scoping review synthesizes evidence published between 2001 and 2023 across eight LMICs (Bangladesh, Sri Lanka, Philippines, Kenya, Ghana, Ethiopia, Rwanda, Peru) to examine how urban and peri-urban food environments shape dietary behaviors and nutrition outcomes.

**Methods:**

Guided by a conceptual framework encompassing nine key food environment dimensions—availability, prices, marketing and regulation, vendor and product properties, accessibility, affordability, desirability, convenience, and sustainability—we analyzed descriptive, associative, and intervention studies. We searched Scopus and Web of Science, identified 1,609 records, and included 251 studies in the review.

**Results:**

As might be expected in a growing body of evidence, most research is descriptive, with limited causal or intervention-based evidence. Studies frequently focus on characteristics of informal vendors, sociocultural factors influencing shifts in dietary choice toward unhealthier options, and the proliferation of ultra-processed foods, especially near schools and in informal markets. Associations between food environment and nutrition outcomes, such as elevated BMI and overweight, are often linked to supermarket and fast-food access, though these relationships are frequently confounded by socioeconomic variables. Methodological inconsistencies in defining and measuring food environment dimensions limit cross-context comparability. Only seven intervention studies were identified, with few demonstrating significant improvements in diet or nutrition.

**Conclusions:**

This review highlights critical evidence gaps in urban food environments in LMICs and underscores the need for standardized measurement and robust evaluations of diet-related interventions. Strengthening this evidence base is essential to inform food policy, urban planning, and public health strategies that promote healthier diets for populations in rapidly urbanizing settings.

**Supplementary Information:**

The online version contains supplementary material available at 10.1186/s12966-026-01884-2.

## Background

Food environments— the combination of the physical, economic, political, and sociocultural surroundings as well as the opportunities and conditions that can influence an individual’s food choice —are undergoing rapid transformation in urban and peri-urban areas of low- and middle-income countries (LMICs) [1]. Over half of the global population now lives in urban areas, a proportion projected to surpass 70% by 2050 [2]. When peri-urban populations are included, this figure climbs to 79%, underscoring a profound demographic shift [3].

Urbanization in LMICs has coincided with the proliferation of fast food, unhealthy ready-to-eat foods, and meals, including ultra-processed foods (UPFs), and sugar-sweetened beverages (SSBs), which are inexpensive, widely accessible, and aggressively marketed [4, 5]. These products meet consumer demand for convenience and affordability but contribute to increasingly unhealthy dietary patterns—high in sugar, salt, additives, and unhealthy fats. Coupled with reduced physical activity, these shifts have fueled rising rates of overweight, obesity, and noncommunicable diseases (NCDs) [6]. Also, the double burden of malnutrition—coexisting undernutrition and obesity—is a growing public health challenge in LMICs. Understanding the evolving nature of urban and peri-urban food environments is essential for designing interventions and policies to foster healthier diets and improve nutrition.

Food environments play a crucial role in shaping consumption choices, as they are the interface where consumers interact with food systems [7]. A commonly used food environment conceptual framework maps external factors (e.g., food availability, prices, vendor and product properties, marketing and regulations) and individual factors (e.g., food accessibility, affordability, convenience, and desirability) that influence consumer choices (Appendix Table 1) [7].

While food environment characteristics impact food purchasing and consumption decisions, the relationships between food environments and food choices are complex and not fully understood [8, 9]. In urban areas of LMICs, food sources range from formal markets (supermarkets, wet markets) to semi-informal (kiosks, mobile stalls) and informal vendors (street hawkers) [3, 10]. In these settings, a substantial share of food is obtained from small-scale and informal vendors, which strongly influences dietary patterns. Given their significant role, more research is needed to better understand the role of informal vendors in shaping urban food choices [10].

The complexity of urban LMIC food environments makes designing effective interventions challenging, with little consensus on how to promote healthier food choices [8]. Most food environment interventions, like food subsidies, unhealthy food taxes, programs to increase healthy food availability, and nutrition labeling, have been implemented in high-income countries (HICs) [11, 12]. Additionally, there is no standardized approach to characterizing food environments. Identifying common indicators could help consolidate measurement approaches, improve comparability across studies, and support frameworks to guide research and policy.

Existing reviews provide important conceptual, methodological, and policy insights into LMIC food environments, but none focus explicitly on urban food environments as a primary analytical lens or use a broader food environment framework; greater focus on informal markets, sociocultural drivers, and methodological and intervention gaps is needed. This creates a clear gap for a review that centers urban contexts, synthesizes urban-specific drivers and outcomes, and draws implications for policy and planning in rapidly urbanizing LMICs. Turner et al., [13] provides a foundational conceptual framework for understanding LMIC food environments across settings, but is largely descriptive and not urban-focused. Westbury et al., [14] synthesized early evidence on associations between urban LMIC food environment characteristics and nutrition outcomes, we extend these syntheses by incorporating more recent evidence, and other food environment dimensions. Framework-focused reviews by Downs et al., [6] do not synthesize evidence specific to urban food environments and nutrition. Toure et al., [15] and Downs and Demmler [11] focus on food environment interventions in LMICs, some of which were implemented in urban or peri-urban settings, but approach the evidence by intervention types rather than by urban dynamics. More recently, Gupta et al., [16] examines research methods and policy relevance of LMIC food environment studies, again including substantial urban evidence without centering on urban-specific dynamics.

Overall, while urban settings are frequently represented in prior reviews, none explicitly synthesizes evidence with urban food environments as the primary focus, highlighting a clear gap that the present review seeks to address. This creates a clear gap for a review that centers urban contexts, synthesizes urban-specific drivers and outcomes, and draws implications for policy and planning in rapidly urbanizing LMICs.

The present review seeks to address this gap by conducting a systematic scoping review of urban and peri-urban food environments across eight low- and middle-income countries in Asia (Philippines, Sri Lanka, Bangladesh), Africa (Kenya, Ghana, Ethiopia, Rwanda), and Latin America (Peru). Scoping reviews are designed to provide a descriptive overview of research trends and to map existing evidence on a given topic [17, 18]. The manuscript is not intended to be representative of all LMICs. Instead, it extracts cross-cutting methodological lessons—such as which food environment dimensions are commonly measured, where indicators converge, and where gaps persist—that may be transferable to other urban LMIC settings.

## Methods

### Overall approach

We conducted a systematic scoping review of urban and peri-urban food environments in eight focal LMICs. A scoping review uses a systematic process to map the coverage of published evidence and to identify evidence gaps, making it particularly relevant for rapidly evolving topics [17, 19]. The Preferred Reporting Items for Systematic Reviews and Meta-Analyses Extension for Scoping Reviews (PRISMA-ScR) checklist and guideline was used in this review [20] which covered a set of eight diverse countries: Philippines, Sri Lanka, Bangladesh, Kenya, Ghana, Ethiopia, Rwanda, and Peru^1^, with varying levels of urbanization. These countries have shared challenges, including increasing prevalence of unhealthy diets and limited access to nutritious foods particularly among low-income urban populations.

### Search strategy and screening

Our search methodology (Appendix Table 2) covered a wide range of terms based on a commonly used food environment framework [7]. We systematically searched Scopus and Web of Science for peer-reviewed studies published in English and Spanish (for Peru) between January 2001 – April 2023 which addressed at least one food environment dimension and used qualitative, quantitative, or mixed-methods designs. The search was conducted on April 13, 2023. Two independent reviewers screened titles and abstracts in the Covidence software (Veritas Health Innovation, Melbourne, Australia, version 2.0) using predefined inclusion and exclusion criteria (Appendix Table 3). Conflicts during screening were resolved through consensus. After initial screening, selected papers underwent full-text review, with exclusions for studies that did not focus on peri-urban or urban areas, had unsuitable designs, lacked country-specific findings, were reviews, or did not report nutrition-related outcomes (e.g., any dietary or nutrition outcome, including measures of dietary quality (e.g. dietary diversity score, minimum acceptable diet, minimum dietary diversity) or anthropometry (e.g. weight-for-height Z scores, weight-for-age Z scores, stunting, wasting)).

### Data charting and analysis

Two reviewers designed data forms and charting variables in an Excel spreadsheet during full text review. They independently extracted the data and discussed any discrepancies. Study characteristics such as target group, publication date, objective, methods, location, data type, and study design were charted. Reviewers classified target groups as vendors, consumers, or both, with further details on vendor characteristics (e.g., ambulatory, stationary, hours, registration status). Location was recorded as capital city, urban area, or peri-urban area. Study designs were categorized as: descriptive (characterizing food environments), observational/inferential (examining food environment-diet and health relationships), evaluations (assessing interventions or exposure to different aspects of food environments), conceptual (proposed frameworks or impact pathways), modeling (use of existing data to model, like Cost of Diet), and laboratory analysis (food safety).

We charted food environment typologies, dimensions, and indicators from each article and categorized food environments into formal, informal, wild, cultivated, and institutional types. External food environment dimensions included availability, prices, vendor/product properties, marketing and regulation, and sustainability. Individual food environment dimensions include accessibility, affordability, convenience, and desirability [7, 21]. Urban interventions in food environments that measured diet (e.g., dietary diversity) and nutrition outcomes (e.g. anthropometry) were also charted. We organized and analyzed data in Excel and R (version 4.2.2). R was used to create bubble plots to visualize data on the number of papers published over time by dimension and by country.

## Results

### Characteristics of included articles

**Fig. 1:**
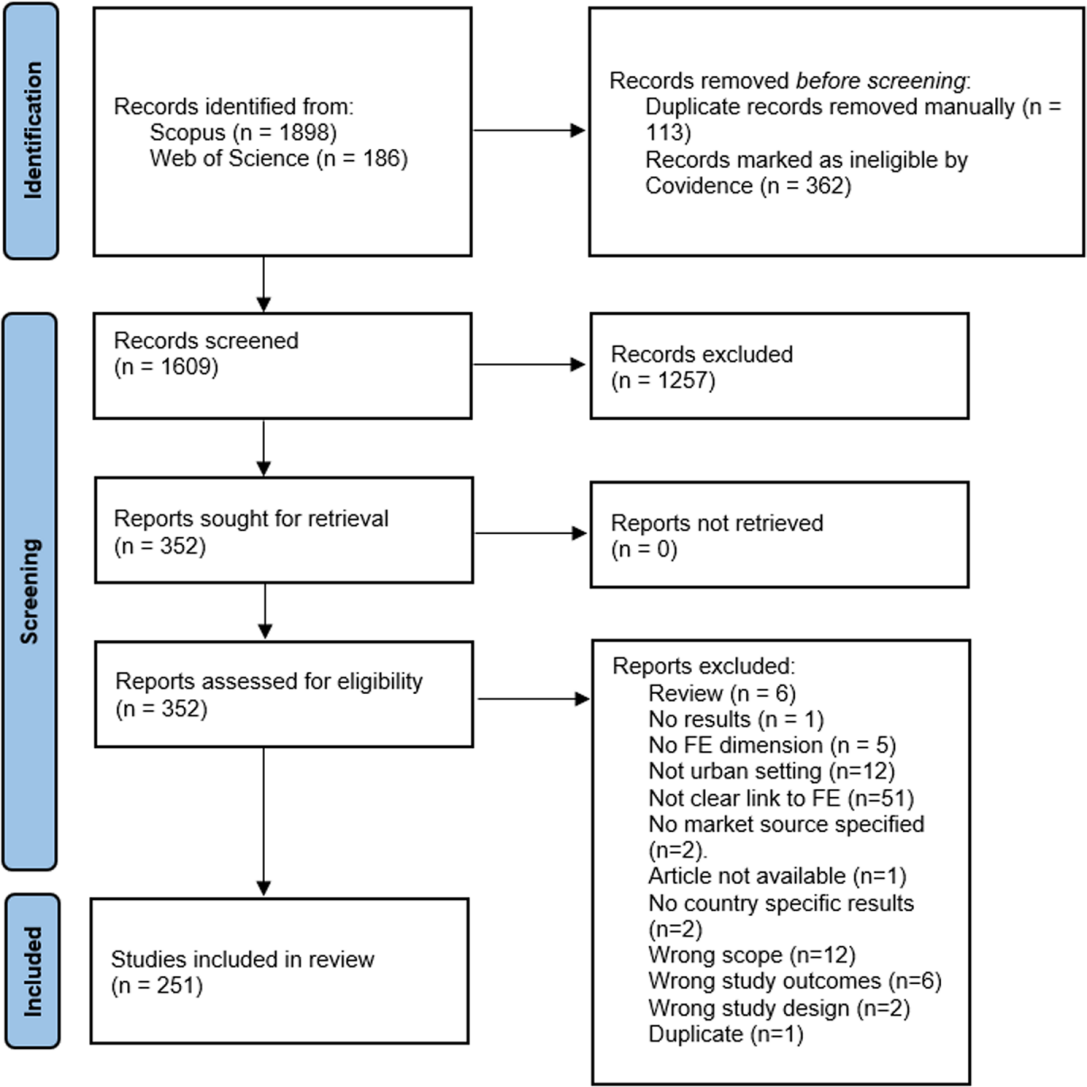
Preferred Reporting Items for Systematic Reviews and Meta-analyses (PRISMA) flow diagram

Our review identified 1,609 titles. After duplicates were removed, abstracts were retrieved from Scopus and Web of Science. After abstract screening, 352 full-text articles were assessed for eligibility. After full text review, 251 studies were extracted and included in our review and 101 articles were excluded (Fig. 1).

Research on urban food environments has been expanding, with 70% of articles published since 2016 (*n* = 176), compared to 19% (*n* = 48) between 2009 and 2015 and 4% (*n* = 11) between 2001 and 2008. The average annual number of publications grew from 2 (2001–2008) to 7 (2009–2016) to 25 (2016–2022).

**Fig. 2:**
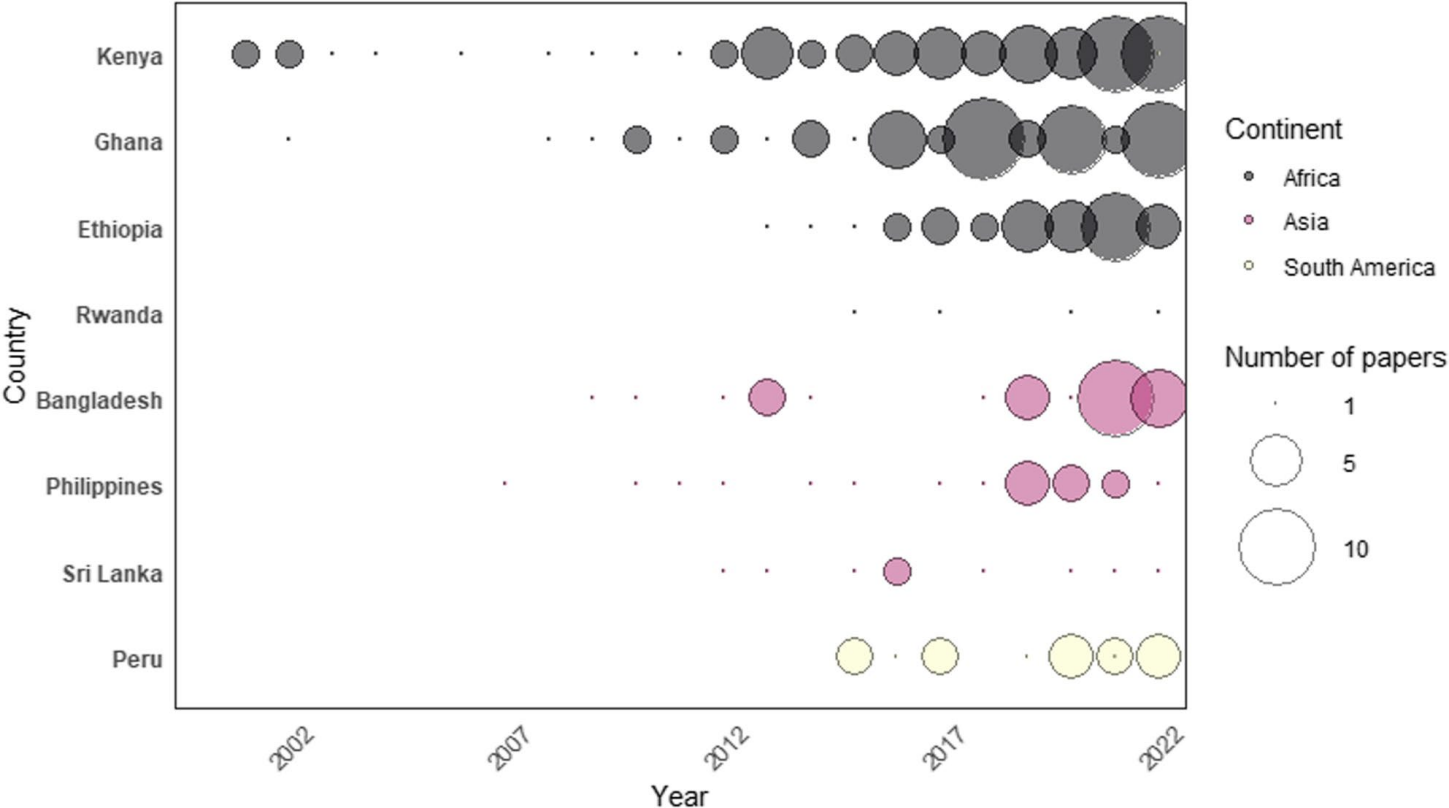
Country-level distribution of urban food environment studies (2000–2022)*. Note: The review surveyed papers through April 2023. Figure 2 presents articles published through December 2022 to better depict yearly trends

Among the eight countries reviewed, Kenya had the most studies (*n* = 72), followed by Ghana (*n* = 56), Ethiopia (*n* = 38), Bangladesh (*n* = 32), Peru (*n* = 22), Philippines (*n* = 18), Sri Lanka (*n* = 9), and Rwanda (*n* = 4) (Fig. 2). The lack of evidence from Sri Lanka and Rwanda may be due to lower urbanization (19% and 18%, respectively).

Among the studies we reviewed, 38% focused on vendors, 57% on consumers, 4% assessed food samples, and 2% did not specify a target group. Most research was conducted in capital cities (57%), 45% covered other urban areas, 2% focused on peri-urban regions and two studies examined an urban-rural continuum.

Most reviewed studies (63%) described food environments, while 15% used observational or inferential methods to examine food environment-diet and health relationships. The remaining studies reported on modeling techniques (8%), conceptual frameworks (6%), evaluations (5%), or laboratory analyses (4%). Most research employed quantitative methods (69%), followed by qualitative (18%) and mixed methods (12%). Nearly all studies (95%) collected single cross-sectional data, with only 5% (*n* = 12) using repeated cross-sectional, panel, or longitudinal data.

### Dimensions and typologies of urban food environments: an overview of the evidence

**Fig. 3:**
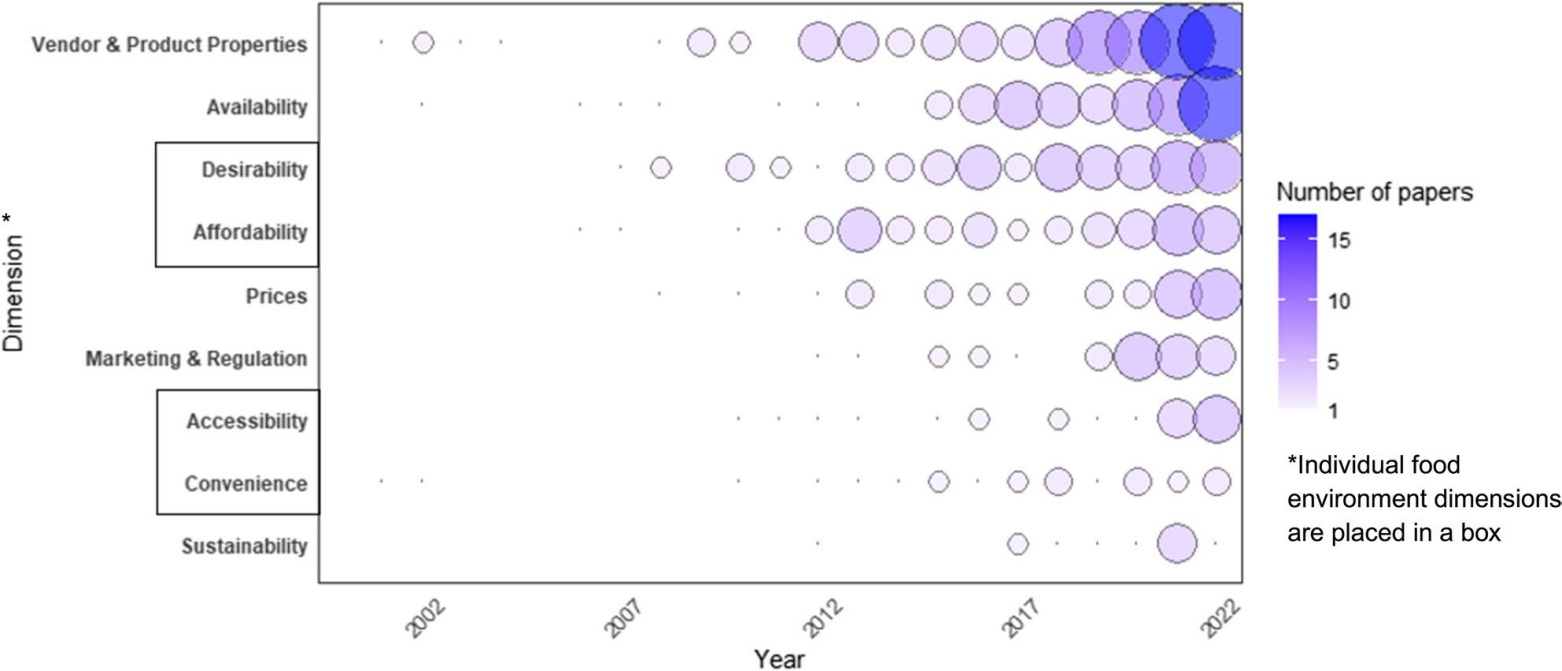
Coverage of food environment dimensions in the peer-reviewed literature (2000–2022)

Vendor and product properties were the most frequently studied food environment dimensions across countries (Fig. 3). 45% of papers (*n* = 112) examined vendor and product property aspects—the majority of which were focused on food safety (71%), followed by availability (28%) and desirability (26%). Food affordability (22%), prices (15%), marketing and regulation (12%), accessibility (10%), and convenience (10%) received moderate attention. Sustainability (5%) was the least covered topic. Studies published before 2010 mostly addressed vendor/product properties and desirability, but subsequent research (2010–2022) expanded to other dimensions (Fig. 3).

Out of the studies we reviewed, most studies focused on formal food environments (57%) such as supermarkets and restaurants, and informal food environments (51%) including wet markets, street vendors, food stands, and kiosks. Institutional settings (e.g., schools, hospitals) and cultivated food environments (e.g., urban agriculture) were each addressed only in 5% of studies, while wild food environments appeared in only 2% (e.g., bushmeat trade in urban Amazonia).

We had originally planned to categorize informal vendors into sub-types (e.g., street vendors, markets), but most papers did not provide a sufficient level of detail to differentiate between types. Most informal vendors were referred to as street vendors, with limited details on mobility, vending schedules or locations, registration status, or the size and diversity of their inventory.

### Description of urban studies by food environment dimension

**Fig. 4:**
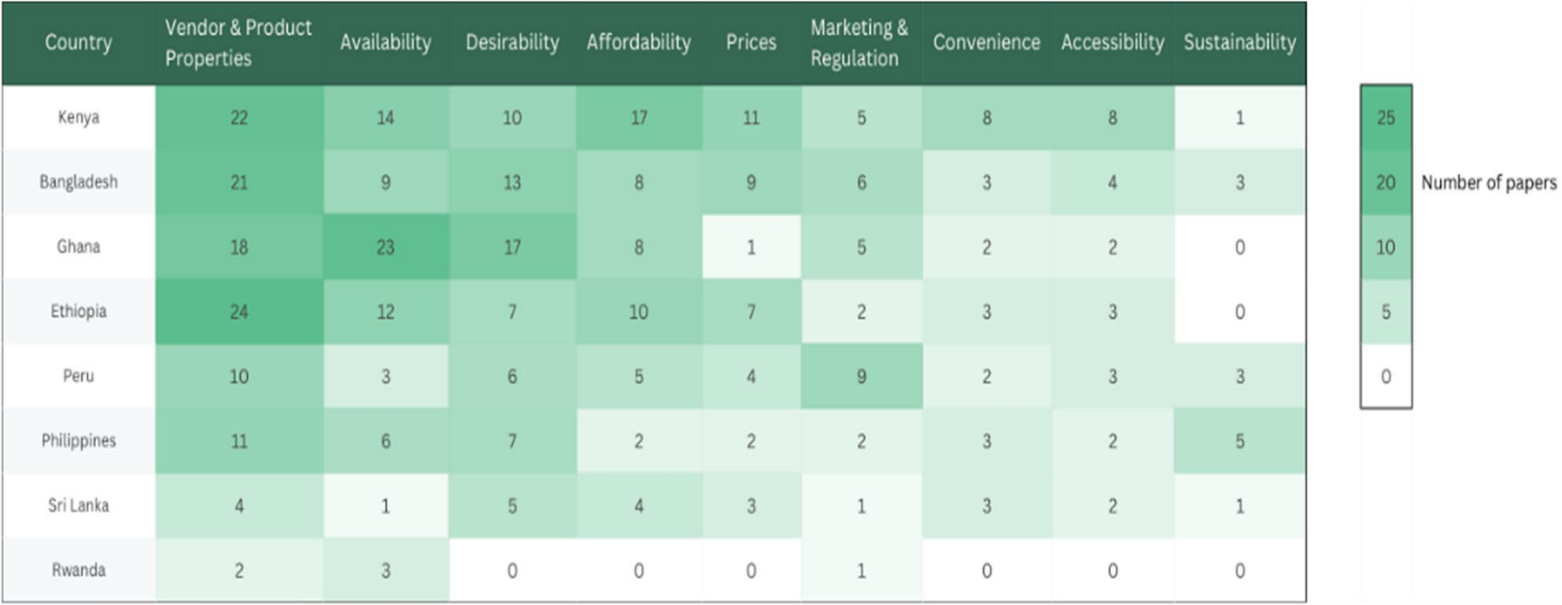
Geographic distribution of food environment research by dimension

Overall, the literature focused on consumer-facing and market-level food environment factors, with limited attention to broader structural and policy-level determinants. Vendor and product properties, availability, and desirability were the most frequently studied food environment dimensions across countries with Bangladesh, Ethiopia, Ghana, and Kenya contributing the most evidence on those areas (Fig. 4). By contrast, dimensions such as sustainability, marketing and regulation, and convenience were less studied, particularly in countries like Rwanda, Sri Lanka, and the Philippines.

We first present external dimensions, then individual-level factors, each ordered by how extensively they have been studied.

#### External dimensions of food environments

##### Vendor and product properties

Residents of informal settlements rely more heavily on street vendors and kiosks than on supermarkets for food purchases [22, 23]. Vendors often target schools, as observed in Kenya [24], and adjust their sales practices in response to consumer demand and regulatory enforcement, as documented in Bangladesh [23]. Food safety was the most commonly reported concern across all countries. Urban vendors typically operate with poor hygiene practices, limited access to water and sanitation facilities, and low levels of food safety knowledge [25–35]. Many studies reported low adherence to food safety guidelines [36–45], and reported contamination in rice [46], vegetables [47, 48], meat and fish (Bangladesh) [49, 50], infant and young child complementary foods (Bangladesh) [50], and milk (Kenya) [27, 29, 31, 51, 52]. Street vendors face particular challenges, including the lack of regulatory protections and the risks associated with informal vending [23, 53].

##### Availability

The widespread availability of UPFs was observed across countries, with the most evidence from Ghana (*n* = 23) [54–58], where the availability of unhealthy foods in and around schools influences children’s dietary behaviors and weight status. These environments reflect broader urban inequalities, with limited access to nutritious options in poorer areas, and highlight the need for policy and infrastructure improvements to support healthier school food environments [59–62]. Perceived availability of healthy options is high in Addis Ababa, but food affordability differs by socioeconomic status with limited access to nutritious diets for poorer households [63]. Evening street vending plays a critical role in expanding food availability, especially for low-income and working-class individuals who rely on affordable, ready-to-eat meals after formal outlets close [64]. In Kenya and Ghana, informal vendors are a vital source for affordable and accessible meals for the poor [65–68], however, the availability of healthy options to the poor was constrained by cost and safety concerns [34, 57, 69]. Rwanda’s open-air markets offered diversity but lacked indigenous crops [70], a concern because indigenous foods in Rwanda are often richer in key micronutrients (e.g., iron, vitamin A, and vitamin C) than commonly consumed non-indigenous staples, and their limited availability may limit dietary quality despite apparent food diversity. Limited urban space generally reduces the availability of cultivated and wild foods, underscoring the need for innovative solutions to improve access to diverse diets [71].

##### Marketing and regulation

Marketing of unhealthy foods is widespread across urban food environments in the eight-country sample. In Ghana and Kenya, nearly half of advertisements in informal markets promoted SSBs, and 29% promoted alcohol [57]. In Peru, the Philippines, and Ethiopia, unhealthy foods were heavily marketed and sold near schools [72–75]. In Addis Ababa, two-thirds of adolescents reported using pocket money to purchase fried foods, sweets, and SSBs [75].

##### Prices

All countries except Rwanda reported on food prices. Informal markets were generally more affordable and attracted lower-income consumers than formal markets [27, 76–78]. In Peru, healthier foods like salad and fruit were more expensive than other restaurant offerings [79], while in Kenya, unaffordable prices of animal-source foods limited consumption [80]. Across contexts, higher food prices were associated with lower diet quality and reduced animal-source food demand [76, 80].

##### Sustainability

Sustainability as a factor in urban food environments remains under-researched, despite the environmental impacts of dietary choices and supply chains. Countries with most research articles on sustainability were the Philippines (*n* = 5), followed by Bangladesh and Peru with three studies each. Filipino urban consumers showed greater interest in sustainable food choices than rural households [81]. Participatory mapping was used to assess food safety risks like exposure to contaminated water impacting food sold in Kenya’s informal settlements [82]. A study in Lima, Peru, found that shifting towards plant-based diets could significantly reduce carbon footprint [83].

#### Individual factors related to food environments

##### Affordability

Affordability is a key factor influencing diet choices in urban and peri-urban areas. In Ethiopia, wealthier households more commonly consume nutritious foods such as vegetables, fruit, and animal-source foods than poorer households, underscoring the role of income in shaping food choices [63]. Households in Ethiopia and Sri Lanka cope with rising food costs by reducing meal frequency or substituting nutritious foods with cheaper, energy-dense (and often nutrient-poor) alternatives [84, 85]. Similarly, studies from Nigeria and Ghana indicate that lower-income mothers and adolescents are more likely to increase consumption of UPFs due to their lower cost relative to healthier options [86, 87].

Despite a willingness to pay slightly more for healthier options in Kenyan poor urban settlements [88], demand for safe or fortified foods remains low, particularly when prices are higher. While urban Peruvians showed interest in community kitchen meals that include fruits and vegetables and were willing to pay modestly higher prices for healthier options [89], studies from Kenya found limited willingness to pay for safe vegetables [90–92] or fortified products [93], especially in traditional markets [94]. These findings highlight tension between health aspirations and economic constraints in shaping food choices in urban food environments.

##### Accessibility

Physical accessibility of food outlets strongly influences food acquisition behaviors in urban and peri-urban areas, often outweighing nutritional considerations. In Kenya, both unhealthy and nutritious foods are physically accessible to consumers across income levels [95–100]. Traditional markets, urban agriculture, and milk dispensing machines are key sources of healthier foods such as indigenous vegetables and fresh milk. In contrast, street food vendors and non-home-prepared food outlets predominantly offer less healthy, energy-dense, and processed foods that contribute to poor dietary quality [78, 101–105]. In Bangladesh and Peru, healthy foods are less accessible than unhealthy options due to higher costs, limited availability, and weak promotion, limiting uptake among low-income groups, disabled migrants, and schoolchildren [106–109]. Accessibility strongly influences food choice and whether basic food needs were met for adolescents, low-income households and disabled migrants [110–112].

Own production could be a way to increase access to healthy foods, especially for fresh vegetables. In Kenya, sack gardening reduced food insecurity, reduced grocery expenditures, and increased consumption of indigenous vegetables but did not significantly improve overall dietary diversity due to production constraints [113]. In contrast, home gardening in the Philippines was associated with greater vegetable consumption and improved dietary diversity among preschool children [114], suggesting that the impact of local food production on diets may vary by context.

##### Convenience

Convenience strongly influences food purchasing time spent on food preparation in urban LMICs, particularly among low-income households who often prioritize time-saving options over nutritional quality. Across countries such as Kenya, Bangladesh, and Ghana, low-income households prefer the convenience of prepared street foods rather than purchasing ingredients from supermarkets and cooking meals, especially where time-constraints are severe [88, 98, 101, 115, 116].

In Ethiopia, Ghana, Bangladesh, and Sri Lanka, studies showed that urban consumers often perceive home cooking as too time-consuming. As a result, many households rely on ready-to-eat foods or meals sold by street vendors [66, 107, 109, 117, 118]. These ready-made options sold by street vendors can serve as important sources of nutrients for some urban households, as documented in Kenya [104]. For other urban dwellers, such as in Kenya and Ethiopia, supermarkets offer similar convenience by providing access to UPFs, which are often preferred over healthier, unprocessed foods typically sold in traditional retail outlets [105, 119, 120].

##### Desirability

Food choices in urban LMICs are shaped by sociocultural factors, including nutrition knowledge, taste preferences, and social norms. While some consumers make decisions based on health awareness or religious or cultural beliefs, others consider food appearance, vendor interactions, and convenience.

In urban Kenya, Ghana, and Sri Lanka, consumers’ food choices are influenced by nutrition knowledge [121], social environments such as family culture and vendor sociability [34, 122] and religious beliefs [117]. In Ethiopia, food taboos during pregnancy affected dietary choices [123, 124]. Physical appearance of food influenced purchasing decisions in Ghana, Kenya, and Sri Lanka [69, 125, 126] while taste preferences were a major driver in the Philippines and Kenya [34, 77, 80, 93, 127, 128]. Studies from Peru and the Philippines also reported frequent consumption of UPFs and limited awareness of their health impacts [129–131].

### Relationships between food environments and dietary and health outcomes

Thirty-two studies analyzed links between food environment components and dietary outcomes, primarily using cross-sectional quantitative methods. Evidence focused on four food environment dimensions - desirability, marketing/regulation, vendor and product properties, and accessibility.

Under desirability, two studies from Ethiopia found that nutrition and food safety knowledge were associated with healthier diets, while food taboos—such as avoiding meat during pregnancy—restricted consumption of certain nutritious foods [123]. Additionally, market disruptions caused by COVID-19 raised prices of staples, pulses, fruits, vegetables, and animal-source foods, leading to reduced dietary diversity [132].

Vendor and product properties, along with accessibility, shape dietary and health outcomes, but the direction of effects remains mixed and context-specific. Shopping at supermarkets in Kenya and Ethiopia was associated with higher household consumption of UPFs, lower intake of fresh foods, and increased adult body mass index (BMI), but also with improved child growth indicators such as height-for-age, weight-for-age, and weight-for-height Z-scores [119, 133]. Differences in the nutritional quality of restaurant food—particularly high sodium content—in low-income neighborhoods explain up to 15% of the socioeconomic gradient in obesity among women in Lima, Peru [134].

No associations were reported between availability, prices, affordability, sustainability, convenience, and diet-related outcomes in the reviewed literature. Most studies lacked an explicit conceptual framework, or assumptions regarding the direction of effects of drivers of food consumption. Some studies applied behavioral theories to explore consumer decision-making around food choices [135, 136], while others examined factors like service quality and trust in shaping consumer behavior. For example, supermarkets were preferred for one-stop shopping, variety, and lower prices (for UPFs) while kiosks were preferable for social interaction and ease of access in Kenya [120].

#### Evidence from longitudinal data

Longitudinal studies highlight how evolving food environments and external shocks shape dietary and health outcomes over time in urban LMICs. Repeated measures captured dynamic trends in food environments and nutrition. In Kenya, increased access to modern food outlets was associated with improved child growth (height- and weight-for-age) and higher adult BMI [133]. In Sri Lanka, rising household incomes—rather than drops in fruit prices—drove increased fruit consumption between 1981 and 2011 [137].

External shocks also significantly impacted urban food environments. In Kenya, COVID-19 restrictions disrupted markets and vendors and reduced dairy demand [138–140]. Post-election violence in Kenya doubled staple food prices, worsening food insecurity in urban slums [141]. In Ethiopia, rising cereal prices due to global commodity price shocks led to fewer meals and increased reliance on less preferred foods among both urban and rural populations [85].

### Evaluations of interventions, programs, and policies in urban food environments

Only seven studies, mostly from Kenya, assessed impacts of food environment interventions. Regulatory measures, such as lockdowns and market restrictions, disrupted food access and affordability [142, 143]. However, informal vendors often bypassed costly compliance requirements, resulting in potential health hazards for both vendors and consumers (Table 1). Another study showed that while food safety interventions improved vendor knowledge, vendors faced practical barriers including shortages of refrigerators and separate utensils needed to manage raw and cooked foods safely. Vouchers were more effective in improving household dietary diversity and food security in urban than rural areas due to stronger market capacity and consumer preference for flexible spending in urban areas. A gardening intervention in urban informal settlements in Kenya improved food security, but resource and coordination challenges limited adoption. Although evidence on food environment interventions is limited, food environments in low-income urban settings are shaped by regulatory constraints, market adaptability, and consumer preferences, underscoring the need for context-specific interventions.

**Table 1:** Evaluations of interventions, regulations, and policies in urban food environments in the eight countries (*n* = 7) 2001–2023

Randomized controlled trial	Location	Population	Intervention	Results
No Kimani-Murage 2022 (Qualitative) [143]	Kenya	Consumers	Regulatory: market restriction measures such as social distancing, curfew, and lockdown on food systems.	Market and mobility restrictions resulted in disruptions in livelihoods, affordability of food, food supply chain and limited availability and access to affordable, safe, adequate, and nutritious food. Some households used urban farming to cope.
No Lelea 2023 (Qualitative) [142]	Kenya	Vendors	Regulatory: Government legal notice banning the sale of raw milk directly to consumers	Informal vendors frequently bypassed public health milk regulations because these rules hindered market operations. For example, vendors resisted switching milk transport containers due to associated higher costs.
No Lagerkvist and Okello 2016 (Quantitative) [92]	Kenya	Consumers	Food safety intervention: consumer willingness to buy through bidding process at market level for vegetables (kale) with enhanced levels of food safety and hygiene. Vegetables (kale) sold at sales stands were safely produced, transported, and managed along the supply chain. Two groups recruited for the bidding: a treatment group who approached the sales stand, and control group who purchased vegetables from another sales point within the same market.	Consumers with greater perceived control over their own food choices experience less regret from purchasing unhealthy foods. Similarly, people with little control over their diets feel greater regret, encouraging healthier choices. Food safety policies should consider psychological factors to shape consumer behavior effectively.
Yes Anyango 2021 (Quantitative) [144]	Kenya	Smallholder dairy farmers (Vendors)	Food safety intervention: Intervention group: training on usage of aflatoxin binder. Control group : no training. All farmers visited biweekly (3 months).	Intervention group had significantly lower aflatoxin (AFM1) concentrations in raw milk over the duration of study. Control group participants were more likely to have milk with AFM1 levels exceeding regulatory limit compared to the intervention. Farmers in the intervention group perceived improvements in milk yield and cow health and appetite.
No Donkor 2009 (Quantitative) [145]	Ghana	Street food vendors	Food safety intervention: one-day risk communication workshop and training for food vendors on food handling using the WHO keys, including topics on food and personal hygiene, environmental and food safety, transmission of food-borne infections, control of foodborne infections and others.	Assessment of the food safety training showed that 68% of the vendors had acquired knowledge from the workshop and were putting it into practice. Lack of food safety equipment was a major barrier to changing behaviors to safer handling practices among vendors.
No Michelson 2012 (Quantitative) [146]	Kenya	Consumers in informal urban settlements, consumers in rural areas	Voucher intervention: food voucher for work in urban informal settlement Mathare (1,000 Kenyan shillings monthly for 3 months), comparative site in Makueni (rural area) of voucher-for-work (150 Kenyan shillings a day) for 10 days per month over three months.	Analysis of existing voucher programs in urban and rural sites; and assessment of market capacity and household modality preferences to determine the suitability of cash vouchers in these contexts. Findings indicated the urban setting responded better to vouchers than surveyed rural areas, and urban households preferred the flexibility of cash or vouchers over food transfers.
No [1] (Mixed-methods, pre-post design) Zivkovic 2022 [71]	Kenya	Consumers in urban informal settlements	Urban gardening program: Training and agricultural inputs to urban sack gardens (no comparison).	Suggestive evidence indicates reduced household food insecurity and post-intervention. Key barriers included insufficient inputs, challenges of group communication. Facilitators included positive feedback, group teamwork, self-sufficiency, and preferences for sack garden vegetables (better quality, no pesticides) over market vegetables.

Note: Regulations and policies are highlighted in red, other intervention types are highlighted in white

### Measuring food environments: key indicators across dimensions

We reviewed a range of studies to assess the coherence of measurement approaches. A wide array of indicators was used for vendor and product properties, marketing and regulation, desirability, and affordability. In contrast, measures for availability, prices, accessibility, and convenience converged on a few common indicators (Appendix Table 4). Sustainability remains an underexplored dimension with no standardized indicators used across contexts.

*Availability* was assessed by categorizing foods offered at various outlets—like schools, markets, street vendors, and restaurants. Indicators measured the presence of healthy and unhealthy foods. Studies used different indicators to measure availability—for example, a study in Kenya applied a healthy–unhealthy food availability score [95], whereas another study used a Market Level Diversity Score [88] and a third study categorized foods by processing level (unprocessed, processed, or ultra-processed) [147].

Food costs and household food expenditures captured *prices*, comparing changes over time or price differences between food outlets. Prices and affordability dimensions often overlapped - with prices used as an input in the definition of affordability. Food prices from various outlets were collected across multiple studies to capture the cost of specific food items. The price elasticity of demand and the effects of changing prices on household welfare or consumption were also examined [148].

*Accessibility* was commonly measured by distance and travel time from a household to food outlets or vendors, often using walking or driving distances. Spatial mapping helped census food outlets, determining their density per square kilometer [112, 149]. For instance, a study in Peru identified food outlets within a 20-minute walk from households [112].

*Convenience* was assessed by the time required to purchase and prepare food, influencing outlet choice. Measures included street food consumption for convenience, as well as the use of home delivery and online food purchases.

Various indicators assessed *vendor and product properties*, with food safety measured in all countries except Peru. This included knowledge, attitudes, food safety practices, contaminant levels in market foods, product characteristics (e.g., organic certification, sustainability, brand, origin), and street vendor conditions. Vendor typologies were reported in all countries.

*Affordability* was measured using tools varying in complexity. One study employed a three-question survey (amount spent on products, quantity purchased, total food expenditure) [80], while another used a single question ascertaining whether food was fully, partially, or not at all affordable [150]. Other studies assessed willingness-to-pay, perceptions of affordability of foods for low-income households, and household food expenditures by recall.

*Desirability* measures evaluated sensory aspects (taste, smell, color) and nutritional quality, as well as environmental, religious, and cultural values influencing food choices. These measures also captured personal preferences, beliefs, attitudes, and factors that drive or hinder food consumption, including shifts toward ultra-processed packaged foods. For example, methods such as photovoice were used to document perceptions of healthy eating and food safety [69, 91].

## Discussion

This systematic scoping review characterized research trends, interventions, and measurement approaches to understand the links between urban and peri-urban food environments and dietary outcomes across eight LMICs in Asia, Africa, and Latin America. In the eight countries studied, urban food environments have been undergoing rapid transformation, marked by increased availability and consumption of inexpensive ultra-processed foods and sugar-sweetened beverages. Despite these rapid changes, research on food environments and how they affect dietary choices and food consumption remains largely descriptive and fragmented, as might be expected in an incipient but growing body of literature, with inconsistent measurement methods and limited evaluations of program or policy impacts.

Key gaps include the absence of robust conceptual frameworks, limited use of rigorous study designs suitable to establishing causal relationships between food environments and diet and nutrition outcomes, and minimal evidence on how different food environment dimensions interact to shape dietary patterns and consumer behavior. The studies that presented conceptual frameworks summarized findings from cross-sectional data offering limited insights into the direction of effects of studied indicators. While some studies explored associations between factors such as nutrition knowledge, market disruptions, and fast-food expansion, there is still insufficient understanding of the drivers of change, or of which populations are most affected.

Measurement challenges are particularly acute. Many studies rely on small or poorly described samples, non-standardized indicators, and unclear conceptual frameworks. While progress is being made in standardizing tools for dimensions such as food prices, availability, and accessibility, further efforts are needed to harmonize methods for assessing household and individual dietary choices, purchasing patterns, and drivers of food choice. Opportunities for improvement include developing standard vendor typologies and refining tools for measuring accessibility, availability, and affordability. Similarly, work is needed for incorporating geospatial methods—drawing on resources like the Food Environment Toolbox [151] and market-focused tools [152] to support more robust and comparable research approaches.

The informal nature of many urban food environments—such as street vendors, wet markets, and small retail outlets—adds complexity to mapping and analysis. Informal outlets play a crucial role in LMICs, yet their density and diversity make it difficult to characterize urban food environments in a way that supports system analysis. Moreover, the lack of detailed descriptive information on the landscape of informal urban food environments presents a limitation to cross-study comparisons and hinders the development of tailored interventions. Studies often treat urban, peri-urban, and rural areas as distinct, overlooking their interconnectedness within food systems. Poorly defined geographic boundaries and inconsistent definitions of urbanicity further hinder understanding, especially in informal settlements where disparities in health and nutrition outcomes are pronounced. Vulnerable groups, including urban refugees, may face additional barriers to food access, underscoring the need for targeted sampling and inclusive research designs [153, 154].

Sustainability is an underexplored food environment dimension. Promising initiatives like Intake4Earth offer tools to assess the environmental impacts of dietary choices [155], but more research is needed on environmental hazard risks, reducing the food system’s ecological footprint, and building sustainable value chains for nutrition. Other initiatives aim to expand upon existing food environment frameworks to capture more dimensions as the body of literature grows. For example, a recently revised food environment framework highlights food literacy and agency as additional personal domain dimensions [156]. Further work should aim to improve metrics and validate these proposed dimensions.

Despite growing concerns about urban nutrition, evidence on effective interventions in urban and peri-urban food environments is limited. As food safety is a clear concern and focus of interest in urban LMICs, it could be considered as a distinct component within food environment frameworks. Promising strategies from HICs—such as front-of-pack labeling, healthy food procurement, zoning, and urban agriculture—have yet to be widely evaluated in LMICs [3, 14]. While some Latin American countries have implemented policies like advertising restrictions and sugar reduction, the countries included in this review have not evaluated large-scale nutrition policies.

To support timely and effective interventions, future research must prioritize longitudinal and mixed-method studies to explore temporal relationships between food environments and diet and nutrition outcomes. Causal analyses are also needed to document successful food environment interventions and lessons learned on the impacts of individual or bundles of food environment interventions on improving the healthfulness of available foods and achieving positive impacts on consumer demand and diet quality. These analyses would help to improve existing food environment conceptual frameworks and understanding of the drivers of food choice. Collaboration among researchers, policymakers, and practitioners will be essential to generate actionable evidence and inform policies that reshape food environments for healthier urban populations.

This review has limitations. It included only articles in English (and in Spanish for Peru) and relied on two databases. Consistent with scoping review methodologies, we did not include a formal quality assessment of the articles reviewed. Moreover, the sample of countries was purposively selected and was not representative of regions, limiting generalizability. Although limited in number, the eight countries represent substantial diversity in regions, urban forms, and food system structures—spanning sub-Saharan Africa, South Asia, and Latin America; megacities, secondary cities, and informal settlements; and formal retail, informal markets, and street food environments. This heterogeneity supports broader inference about common challenges and opportunities in urban food environment research across LMICs. Consistent with the purpose of a scoping review, the aim is not comprehensive geographic coverage but to map the range of existing evidence, approaches, and gaps across diverse contexts where research exists, thereby informing future research priorities and methodological development. By highlighting where evidence is concentrated and where food environment dimensions remain underexplored, the review also outlines key areas for future work in urban LMIC contexts. Our focused approach enabled detailed, place-based analyses, offering valuable information from case studies and contributes to filling evidence gaps in under-researched urban settings in eight countries, providing a foundation for future comparative or regionally representative research.

## Conclusions

This systematic scoping review of urban and peri-urban food environments in eight LMICs highlights how external and individual-level factors shape diets and nutrition. Informal vendors play a vital role in food access in countries such as Kenya, Bangladesh, and Ethiopia. In Peru, Philippines, and Ghana, ultra-processed foods were increasingly marketed near schools. Across all countries, affordability, food safety concerns, and limited nutrition awareness constrained healthier food choices, even in contexts where food diversity exists.

Our findings show that food choices are influenced by income, convenience, proximity, and sociocultural norms. In Ethiopia, Ghana, and Sri Lanka, economic factors and time constraints often lead households to rely on cheaper, less nutritious foods. Evidence linking food environments to nutrition outcomes is growing but remains limited. The few intervention studies—primarily from Kenya—suggest that market-based approaches such as vouchers and urban gardening can improve food security, though regulatory and food safety efforts face implementation challenges. Future research should prioritize rigorous study designs, standardized tools, and alignment with urban nutrition policy to inform context-specific, scalable solutions.

## Supplementary Information

Supplementary Material 1.

## Data Availability

The datasets used and/or analysed during the current study are available from the corresponding author on reasonable request.

## Abbreviations

AFM1: Aflatoxin
BMI: Body mass index
COVID-19: Coronavirus disease of 2019
HIC: High income country
LMIC: Low- and middle-income country
NCD: Non-communicable disease
PRISMA: Preferred Reporting Items for Systematic Reviews and Meta-analyses
PRISMA-ScR: Preferred Reporting Items for Systematic Reviews and Meta-Analyses—Extension for Scoping Reviews
SSB: Sugar-sweetened beverage
UPF: Ultra-processed food

## Footnotes

1 These countries are targeted under the CGIAR research initiative ‘Resilient Cities through Sustainable Urban and Peri-urban Agrifood Systems’ and were selected through a participatory design process involving stakeholders and technical experts from the Consultative Group for International Agricultural Research (CGIAR), World Vegetable Center, RUAF, and an advisory group with the World Bank, FAO, donor agencies, universities and global city networks (Milan Urban Food Policy Pact, International Council for Local Environmental Initiatives (ICLEI), Cities Climate Leadership Group (C40)). Funding for this work was provided by the same initiative.
