# Supplementary material for: A systematic scoping review of urban food environment research, interventions, and measurement approaches in eight low- and middle-income countries
Source: Int J Behav Nutr Phys Act. 2026 Mar 5;23:39. doi: 10.1186/s12966-026-01884-2 (PMC13097719; doi:10.1186/s12966-026-01884-2)
Supplement: Supplementary file 1 — Supplementary Material 1. [file 12966_2026_1884_MOESM1_ESM.docx]

Appendix

Table 1: Food environment dimensions adapted from Turner 2018 and Constantinides 2020

| **Dimension** | **Definition** |
| --- | --- |
| Availability | The presence of a food or food product |
| Prices | Value of food products in monetary terms |
| Vendor and product properties | Vendor properties (hours of operation, typology), Product properties (food quality, safety of food, packaging) |
| Marketing and regulation | Advertising, labeling, policies, branding |
| Accessibility | Distance to markets, mobility, space, transportation to vendors |
| Affordability | Purchasing power or food prices in relation to a consumer’s income |
| Convenience | Time and effort to prepare, cook, and consume foods |
| Desirability | Consumer’s preferences, tastes, culture, knowledge, skills related to food |
| Sustainability | Environmental aspects of food, climate footprint |
| Stability | Seasonal fluctuations affect food availability and affordability |

Table 2: Search methodology

Scopus

| **FE domain** | **Search** |
| --- | --- |
| Overall food environments | TITLE-ABS-KEY ( "food environment*" OR "food desert*" OR "food swamp*" OR "obesogenic environment*" OR "nutrition* environment*" OR "neighborhood environment*" OR "neighbourhood environment" OR "retail* environment*" OR "supermarket*" OR "superstore*" OR " hypermarket*" OR "food mart*" OR "food outlet*" OR "corner shop*" OR “local shop*” OR “general market*” OR "foodscape*" OR "traditional market*" OR "wet market*" OR "open-air market*" OR "open air market*" OR "fruit* vegetable* market*" OR "fruit* and vegetable* market*" OR "vegetable market*" OR "fruit market*" “street market*” OR "food retailer*" OR "food establishment*" OR "grocer*" OR "food chain store*" OR "chain food store*" OR "retail food market*" OR "food retail market*" OR "food shop" OR "fruit* vegetable* shop*" OR "fruit* and vegetable* shop*" OR "vegetable shop*" OR "fruit shop*" OR "greengrocer*" OR "convenience store*" OR "superstore*" OR "super store*" OR "food retail store*" OR "traditional store*" OR "food store*" OR "discount store*" OR "fast food store*" OR "dairy product* store*" OR "small store*" OR "corner store*" OR "co op*" OR "co-op*" OR "food* market" OR "food* markets" OR "restaurant*" OR "take$away*" OR "carry$out*" OR "online retail*" OR "kiosk*" OR "mobile vendor*" OR "farmer$s market*" OR "canteen*" OR "wild food" OR "food gather*" OR "hunt*" OR "forag*" OR "own production" OR "own-production" OR "homestead farm*" OR "garden*" OR "orchard*" OR "cultivated plot*" OR "field*" OR "forest*" OR "food aid" OR "food assistance" OR "food barter*" OR "food sharing" OR "community food" OR "festival*" OR "food remit*" OR "school*" OR "hospital*" OR "prison*" OR "institutional food" OR "institutional meal*" OR "workplace*" OR “food industry” OR “food industries” ) |
| Availability | TITLE-ABS-KEY (“food production” OR "perceive* avail*" OR "food" W/2 "avail*" OR "food shortage*" ) |
| Prices | TITLE-ABS-KEY ( "cost of" near/5 "diet" OR “food price*” OR “food pricing”) |
| Vendor and product properties | TITLE-ABS-KEY ("food vendor*" OR "street vendor*" OR “cycle vendor” OR "food$born* illness” OR "food safety" OR “food qualit*” OR "food process*" OR "food packag*" OR "food shelf-life" OR "food composition" ) |
| Marketing/regulation | TITLE-ABS-KEY ( "food" near/3 "advertis*" OR “marketing” OR “food packag*” OR “food label*” OR “food polic*” OR "food brand*" OR "food sponsor*" OR "food promot*" OR "nutrition polic*" OR "food tax*" OR "food regulat*" OR "sugar tax*" OR "soda tax*" OR ("diet*" AND "social media") OR ("food*" AND "social media") OR ("food*" AND "digital") |
| Sustainability | TITLE-ABS-KEY (“sustainable diet*” OR “sustainable food*”) |
| Stability | TITLE-ABS-KEY ( "food*" W/2 "year round" OR "food*" W/2 "year round" OR "food stab*" OR "food price*" W/2 "fluctuat*" OR "season*" W/2 "food production" OR "season*" W/2 "food avail*") |
| Accessibility | TITLE-ABS-KEY ("perceive* access" OR "food access*" OR "food" W/2 "access*" OR "distance* to market" OR "distance* to food") |
| Affordability | TITLE-ABS-KEY ("perceive* afford*" OR "afford*" OR "food" W/2 "afford*" OR “income variability” OR “food purchas*”) |
| Convenience | TITLE-ABS-KEY ("convenien*" AND "ready-made food*" OR "food deliver*" OR "premade food*" OR "pre-made food*" OR "premade meal*" OR "pre-made meal*" OR "food prepar*" OR "meal prepar*" OR "precook*" OR "pre-cook*" OR “cooking” OR “consump*” OR “food” W/3 “time” OR “food" W/2 "prepar*”) |
| Desirability | TITLE-ABS-KEY ("taste preferenc*" OR "food attitude*" OR “food belief*” OR "food*” W/2 “desir*" OR "food preferenc*" OR "food accept*" OR “taste” OR "food cultur*" OR "food choice*" OR ("food*" AND "consumer behavior*) OR "feeding behavior*" OR "food taboo" OR "feeding practice*") |
| Urban-peri urban | TITLE-ABS-KEY ("urban*" OR "cities" OR "city" OR "slum" OR "slums" OR "peri-urban" OR “peri urban” OR "metropolitan*" OR "metropolis") |
| Ethiopia | TITLE-ABS-KEY ("Ethiopia*" OR “Addis*” OR “Dire Dawa*” OR “Mekelle*” OR “Adama*” OR “Awassa*” OR “Hawassa*”) |
| Bangladesh | TITLE-ABS-KEY ("Bangladesh*” OR “Dhaka*” OR “Chittagong*” OR “Khulna*” OR “Rajshahi*” OR “Comilla*”) |
| Peru | TITLE-ABS-KEY ("Peru*" OR "Lima" OR "Arequipa" OR "Trujillo" OR "Chiclayo" OR "Piura" OR "Iquitos" OR "Cusco") |
| Philippines | TITLE-ABS-KEY ("Quezon city" OR "Manila" OR "Caloocan" OR "Davao" OR "Cebu" OR "Zamboanga" OR "Antipolo") |
| Rwanda | TITLE-ABS-KEY ("Kigali" OR "Gisenyi" OR "Ruhengeri" OR "Butare" OR "Muhanga" OR "Byumba" OR "Cyangugu") |
| Sri Lanka | TITLE-ABS-KEY ("Colombo" OR "Dehiwala" OR "Moratuwa" OR "Sri Jayawardenepura*" OR "Negombo" OR "Kandy" OR "Kalmunai") |
| Ghana | TITLE-ABS-KEY ("Accra" OR "Kumasi" OR "Tamale" OR "Sekondi-Takoradi" OR "Ashaiman" OR "Sunyani" OR "Cape Coast") |
| Kenya | TITLE-ABS-KEY ("Nairobi" OR "Mombasa" OR "Nakuru" OR "Ruiru" OR "Eldoret" OR "Kisumu" OR "Kikuyu") |
| Year | PUBYEAR > 2000 |

Web of Science

| Theme | | Terms |
| --- | --- | --- |
| Food Environment and individual-level factors | Overarching food environment terms | “food environment*” OR “food desert*” OR “food swamp*” OR “obesogenic environment*” OR “nutrition* environment*” OR “neighborhood environment*” OR “neighbourhood environment” OR “retail* environment*” OR “supermarket*” OR “superstore*” OR “ hypermarket*” OR “food mart*” OR “food outlet*” OR “corner shop*” OR “foodscape*” OR “traditional market*” OR “wet market*” OR “open-air market*” OR “open air market*” OR “fruit* vegetable* market*” OR “fruit* and vegetable* market*” OR “vegetable market*” OR “fruit market*” OR “food vendor*” OR “street vendor*” OR “food retailer*” OR “food establishment*” OR “grocer*” OR “food chain store*” OR “chain food store*” OR “retail food market*” OR “food retail market*” OR “food shop” OR “fruit* vegetable* shop*” OR “fruit* and vegetable* shop*” OR “vegetable shop*” OR “fruit shop*” OR “greengrocer*” OR “convenience store*” OR “superstore*” OR “super store*” OR “food retail store*” OR “traditional store*” OR “food store*” OR “discount store*” OR “fast food store*” OR “dairy product* store*” OR “small store*” OR “corner store*” OR “co op*” OR “co-op*” OR “food* market” OR “food* markets” OR “restaurant*” OR “take$away*” OR “carry$out*” OR “online retail*” OR “kiosk*” OR “mobile vendor*” OR “farmer$s market*” OR “canteen*” OR “wild food” OR “food gather*” OR “hunt*” OR “forag*” OR “own production” OR “own-production” OR “homestead farm*” OR “garden*” OR “orchard*” OR “cultivated plot*” OR “field*” OR “forest*” OR “jungle*” OR “food aid” OR “food assistance” OR “food barter*” OR “food sharing” OR “community food” OR “festival*” OR “food remit*” OR “school*” OR “hospital*” OR “prison*” OR “institutional food” OR “institutional meal*” OR “workplace*” OR (“cost of” NEAR/5 “diet”) OR “food$born* illness” OR “food safety” OR (“food” NEAR/3 “advertis*”) OR  “food access” OR “afford*” OR “desir*” OR “food preferenc*” OR “taste preferenc*” OR “food attitude*” OR “food accept*” OR “convenience” OR “perceive* avail*” OR “perceive* afford*” OR “perceive* access” OR (“perception*” NEAR/3 (“avail*” OR “afford*” OR “access*”)) |
|  | (OR) Formal and informal market types |  |
|  | (OR) Wild and cultivated types |  |
|  | (OR) Food aid, services, and kin & community |  |
|  | (OR) Food environment dimensions |  |
|  | (OR) Individual-level factors |  |
| (AND) Food | | “Food*” OR “grain*” OR “cereal*” OR “rice*” OR “potato*” OR “vegetable*” OR “fruit*” OR “leafy green” OR “veg” OR “nut*” OR “legume*” OR “pulse*” OR “bean*” OR “egg*” OR “dairy” OR “dairies” OR “milk*” OR “yogurt*” OR “cheese*” OR “fish*” OR “seafood*” OR “meat*” OR “sugar sweetened beverage*” OR “high fat” OR “high sugar” OR “high salt” OR “diet*” OR “eat” OR “eating” OR “nutr*” OR “snack*” |
| (AND) Context/Setting | | "Sri Lanka*" OR "Philippines" OR "Filipino" |
| (AND) Year | | 2000 - 2022 |

Table 3: Inclusion and exclusion criteria for urban food environment scoping review

|  | **Inclusion criteria** | **Exclusion criteria** |
| --- | --- | --- |
| **Population/ setting** | -Peri-urban and urban areas in one or more of these countries: Ethiopia, Bangladesh, Kenya, Ghana, Rwanda, Peru, Philippines | - Rural settings - Countries other than the ones listed |
| **Scope** | - Features one or more of these FE dimensions: availability, pricing, vendor properties, product properties, marketing, regulation, availability, affordability, convenience, desirability, stability, social forces, food safety - Includes at least one dimension of urban food environments - Was published from January 2001 -April 2023 - Formal or informal food environments, wild or cultivated food environments - Interventions related to at least one of the food environment dimensions | -Studies that increase food availability or affordability at macro level  -Studies to improve crops or barriers to crop productivity that are not related to consumption of target population/s  -Genetic analyses, chemical laboratory testing, field evaluation of invasive species, plant diseases, etc.  -Evaluation of soil quality/characteristics  -Studies of plants as medicine  -Assessed anthropometry, diet or micronutrient intake but not any food environment determinants or dimensions  -Assessed nutritional composition of food items but not environment  -Assessed environmental exposures not related to diets, food/s and or food safety  -Studies focused on one food type or item without considering aspects of the FE |
| **Populations** | -Populations located in urban or peri urban settings, can include combination of rural/urban as long as includes an urban population explicitly | -Studies of migrant populations living elsewhere; nomadic populations, temporary workers or others not primarily residing in urban/peri urban areas |
| **Study design** | Quantitative, qualitative, mixed methods observational studies (cross-sectional, case-control, cohort studies), or experimental studies (randomized controlled trials), or quasi-experimental designs (interrupted time series, regression discontinuity)  -Reviews/systematic reviews | -Editorials, commentaries, opinions, conferences, blogs  -Must be published (not grey literature)  -Exclude validation studies  -Systematic or scoping reviews |

| **Table 4: Indicators across food environment dimensions** | | | | | | | | |
| --- | --- | --- | --- | --- | --- | --- | --- | --- |
| **FE dim** | **Ghana** | **Peru** | **Kenya** | **Bangladesh** | **Ethiopia** | **Sri Lanka** | **Philippines** | **Rwanda** |
| **Availability** | types of street foods available; presence of supermarkets/shopping malls; no. of F&V stands; no. of convenience stores; presence of vendors near schools; types of foods sold; types of vendors; type of food in the market (unprocessed, processed, ultra-processed); presence of edible trees species in urban environment (including home gardens) | Number of food courts; Presence and number of fast food institutions | business characteristics; what food is available; engagement in urban agriculture and urban-based rural agriculture; a place where you eat food and/or beverage from; variety of food sold by community vendors; diversity of porridge ingredients; retail outlet choice; Sources of non-home prepared foods; foods available in environment; Spatial Distribution of Vendors; vendor location; sources of marketed milk; market-level diversity score (MLDS); unhealthy/ healthy food availability score | % Respondents mentioning food unavailable in market; availability of fast foods around schools; Presence of various oils on markets and where they can be purchased (vendor type/source); Market availability of various food items; food stock availability of food items at the household level; Types of fish sold at urban markets; Availability of fast food; Availability of fish or meat; Relatives and neighbors sharing food during lockdown; government food provisions during lockdown; Street vending making products more available; Presence of coca cola in every shop within a 1km radius | Household perceptions on food availability; types of street foods; types of food fed to child; types of food available in the market; type of F&V sold in the market; type of coffee available at different traders; availability of foods near schools and home environment; availability of ultra processed foods in schools ; availability of F&V at home; sources of meat and milk at market and farm | Availability of organic, sustainable, regionally produced food items | Availability of vegetables; Food availability from different vendor outlets; Use of fast foods and fast-food delivery services ; Availability of food and expected shortages | Inventory of food categories and country of origin; Measures of coverage for iron enriched beans and orange fleshed sweet potatoes, Market source for biofortified crops, IBs/OFSP available to households; Number indigenous foods available in markets Availability of biofortified foods |
| **Prices** | Price difference (based on vegetable freshness) | Price of fish, Average price of food products obtained from the Peruvian Statistics Institute, price of salads | Price of chicken product; price of milk purchased; selling price per litre; ATM machine and milk sale; converting local units of reported household purchases to kilogram weight; elasticity of demand; willing to buy yellow maize meal at the same price as their current preferred maize product; vegetable prices; prices of each food item; foods increased or decreased in price | % of the households reporting an increase in food prices; Cost of the food indicative of quality of food; Price of organic foods, bulk palm oil, soybean, super palm oil, and other branded oils; Daily expenditures on food; Local fish prices, perceived changes in fish prices pre vs post COVID; Perceived value/price of restaurant food; Price increases of key foods during COVID-19 lockdown of meat; prices of vegetables; cost of products vended | Average cost of food; Change in vegetable prices; Cost of F&V sold in the market; Changes in prices of staples, pulses, fruit and vegetable and animal sourced foods ; Effect of price change on diets; Market prices; Coffee prices; % increase/decrease in food prices and how it relates to household welfare and consumption | Cost as barrier to eating healthy food | Price and cross price elasticity to understand price sensitivity; Vegetables are cheaper compared to foods sold at supermarkets and malls |  |
| **Vendor and Product Properties** | Food safety KAP; frameworks for governing food safety; access to hygiene facilities; microbial levels in food; storage facilities; type of trader | Product properties; caloric intake, processed and ultra-processed foods knowledge, genomic DNA of fish, amount of critical nutrients (that is, saturated fat, sugar, sodium, trans-fat) and the total energy, nutritional analysis, PDRNI carbohydrate, fat, sodium, conventional or organic products, Vendor properties; questionnaire identifying street vendors located, at departure time, throughout the street in front of the doors of various institutions | Common food safety problems; food safety checklist and direct observation; risks associated with fresh vegetables; quality standards; sanitory practices for food safety; knowledge, attitudes, and practices in food safety; lab testing; trust in information about potential health risks associated with production, handling, and consumption of kale; hygiene awareness, Aflatoxin assays; perceived risk, control, and volition; food safety; hygienic practices; Knowledge of HACCP, milk handling, operation practices; harassment of vendors and the imposition of bribes; product names, ingredients, methods of preparation, and the sex of the vendor; The Food Environments Working Group (FEWG) Framework of the Agriculture and Nutrition for Health academy; sale of leafy greens; propensity for elevating risk of obesity, Vendor typology (operations, affiliations), Practices at production, collection/bulking, processing and transportation; location and condition of informal food stands | Vendor properties: School-based vendors’ knowledge about basic food safety issues and schoolchildren health; Trustworthiness in halal food providers; trustworthiness in halal food items; mapping movement of street vendors throughout the day; Risk perception of liquid milk (related to food safety); Knowledge, attitude, practice of food safety; Use of protective equipment by vendors; Salesmanship and decoration of vendor stalls; privacy of establishment (I prefer the fast food shops that offer privacy); Food handling practice (14 question measure); Extra workers at a vending stall; hygienic practices among food vendors; Reputation/brand/image of a restaurant, service quality: customers who compare expectation and performance after receiving a service, trust in a restaurant, Food hygiene practices of street vendors and restaurant staff before serving foods; Food safety norms; Vendor typology - focus on fast food restaurants; Product properties: locally produced or imported properties; Quality, brand, expiry date, location of origin; food quality (product properties); Average daily sale, daily average profit margin, magnitude of change in sale, magnitude of change in profit margin before and after COVID-19 restrictions; fat and cholesterol content, cleanliness and hygiene (food safety), brand reputation; Total coliform and faecal coliform count; food hazard perception (is food contaminated with bacteria, does food contain pesticide residues); food safety (food hygiene) of fast food vs. home cooked foods | Vendor knowledge and practice of food safety; snack food product shape; flavor and main ingredients; Type of vendor (home delivery, dairy shops, retail outlets); Food quality; type of vendor (farmer or milk shop); Sanitary status of food establishments; Product storage facilities; Type of vendors selling fruit and vegetables within 1km radius; Vendor socio-demographic characteristics; vendor selling times; Food quality (presence of microbes); food safety training of vendors; Hygiene conditions of vendors; Types of vendors for milk and meat available to consumers;  food safety practices (hygienic condition of milk sold in the market; milking procedures at household level; storage procedures at household and market level); food quality (bacterial load) | Diet that includes processed or fried foods; food safety of green leafy vegetables, a staple food; Food safety of rice; Product characteristics such as organic food, sustainable, regionally prodoced food items etc | Testing for presence of microplastics in commercially sold fish; Restaurant attributes of food quality, food safety, service quality, and store atmosphere; Quality - storage, preserving, processing and preparation; Compliance of ambulant food vendors to food safety standards; Product properties such as packed canned tuna in oil or sauce; Attributes of environmental sustainability and food safety; Food safety - screen for the presence of chloramphenicol residues in retailed chicken; Vegetable stand/kiosks and how it offers fresh organic produce; Levels of residual nitrite in skinless native sausage; Reasons for changes in food consumption practices - food quality, compositions (new cuisines to explore); Detect Salmonella spp. from various raw meat and meat products collected from wet markets; Food safety - testing for presence of food-borne parasites in green leafy vegetables | Product characteristics (i.e. sold fresh, flour, etc.); Hygiene indicator bacteria (E.coli, Salmonella), temperature conditions for transport, storage and sale of meat at food outlet; Origin of processed meat, cleanliness of outlet, cleaning frequencies, medical checkups for staff, protective clothes by handlers, personnel safety/hygiene training in meat handling |
| **Marketing/ Regulation** | Food advertisement near schools; packaging; food and nutrition labels | Governance related variables, Knowledge of front labels (octagons) on the containers of processed and ultra-processed products, MINSA guidelines, brand, product category, general communication concept, color, product representation, environment/featured activity, number, and gender of the persons shown in the ad, health claim, values/emotional appeal, gender of the main character, role model/ public figure, public to which it is visually directed, focal point, typography, scales, shape, and contrast, name of fish, FOP labelling, organic vs non organic, 20 COVID measures | Self-regulation; opinions on regulation; List the types of agreements, rules and regulations they had to follow (legislative, private standards etc.). e List the types of sanctions/penalties experienced for not adhering to such agreements; certification, licensing and testing requirements; type of advert | Number of stores with breastmilk substitute promotions (price promotions, free customer gifts); Media advertisements for breastmilk substitutes (online, TV, radio, newspaper, mother online forums); COVID lockdown policies (market closures, trade disruptions, transportation ban, and migratory labor movement restrictions) influence on consumption; Regulations for street vendors (street vending currently not governed by regulations); Influence of cooking shows, TV shows on influencing consumption of fast foods; Advertisement for unhealthy foods (ice cream); billboard and newspaper ads from trans-national food chains; increased use of computers, TV and internet consumption; marketing strategies of fast food chains; Frequency of exposure to TV advertisements; TV ad cues (celebrities, package color, song and music, etc.); appeal of branded snacks and beverages in TV advertisements (taste, nutrition, price, etc.) | Nutrition/health claim labeling; Types of advertisements | Participants use of food labels and brand recognition: Information given on the labels of packaged snacks are trustworthy if it had a brand name that is popular (Likert scale from strongly disagree to strongly agree) | Food advertisements in the school vicinity |  |
| **Sustainability** |  | IUCN 365 Red List of Threatened Species, greenhouse gas emissions. lifecycle method | Environmental justice framework | Concerns about climate change, the importance of mitigation policies and efforts, and environmental and climatic imprint |  |  |  |  |
| **Stability** | Vendor price fluctuations based on seasonality | the stakeholders involved, their relationships, and the functioning of the trade, household food security, food assistance program participation, informal methods of food access | Household Food Insecurity Access Scale (HFIAS) and the Household Food Insecurity Prevalence (HFIAP) tool; dependence on food from different sources, food insecurity coping strategies, and households’ perceptions on importance of own agricultural production; Poverty rate – the percentage of people living below the national income level – + Household Hunger Score (HHS); modified version of a tool developed by USAID FANTA to measure overall dietary diversity as well as the diversity of vegetable consumption amongst households in Kibera; quality, safety, and household food distribution; Household Food Insecurity Access Score; production shortfalls; geographical and temporal profiles; engagement in urban agriculture; share of total household expenditure, total reported energy intake per capita | Seasonality of specific foods |  |  |  |  |
| **Accessibility** | Distance to markets/street foods; | Physical environmental influences "perceived vs observed: neighborhood food outlet census, Recording food outlets’ GPS coordinates, Perceptions of the access, quantity, and quality of foods in the participants’ neighborhood, and perceptions of access, quality, and cost of foods in frequently visited food outlets; neighborhood defined as the area accessible within 20 min of walking from their home; Unmet need for access to food in the household (“Yes” if “Food” is one of the main needs not being met in the household) | What quantity they bought where; spatial mapping; distance to the source of milk and milk products; open-ended questions relating to dietary practices and household decision-making; the number of supermarkets per square kilometer; access to safe foods; a place where you eat and/or drink; foods accessible | % experiencing difficulty accessing food in general; % unable to visit the market due to mobility restrictions; Proximity to fast food vendors; Nearness, accessibility; fast food chains available within a 2km radius from campus | Access to milk retail outlets; Distance to sellers; Time taken to reach vendor/shop |  | Structured interviews were used to collect data on the following topics: household access to foods among others - through Producing, buying and exchanging; Accessibility via mode of transport- walking and driving distance to the stand from household; Access due to curfew and lockdowns |  |
| **Affordability** | Willingness to purchase; ability to purchase; affordability of food baskets; purchasing power; | Willingness to pay, affordability of menu price, affordability of quinoa, Types of food low income households can purchase | Willingness to pay for chicken, porridge, food safety; How much they spent; household income level; factors driving food purchasing; level of income; effect of income level on milk consumption; ‘During the week preceding the survey a) how much did you spend on the following products? b) what was the quantity purchased? c) what was the total expenditure on all foods?‘. Almost Ideal Demand System (AIDS); Household purchasing power; Expenditure levels; Household spends the greatest proportion of their food budget; the quantity of a given staple food affordable per average monthly (1) urban per capita income, (2) per capita GDP, (3) formal sector wage, and (4) services sector value-added per employee, and retail staple food prices deflated by the (5) non-food CPI and (6) GDP deflator | % who can afford to buy food in general; Affordability of food (response options: full, partial, not at all); Types of food low income households can purchase; Purchase of food in urban areas in contrast to rural areas (where respondents moved from, had fresh and more affordable F&V); unaffordability of milk and fish for the poor; incomes; Ability to afford foods during the COVID-19 lockdown; Affordability of fast food produced by trans-national companies and soft drink companies | Household perception on food affordability; Amount of money consumers willing to pay for snack products; willingness to pay (WTP) for pasteurized, whole milk, reduced fat milk; quantity of food purchased; cost of food purchased; Affordability of food baskets; WTP for fortified foods; Food consumption expenditure; Types of food purchased | Perception that instant food is cheaper than fruits, rice and curry; Cooking at home is more costly; Higher prices for organically produced food acts as a barrier for purchase; Price of fruits as an independent variable when modeling demand for fruits | Vegetables on the market are cheaper compared to those sold at supermarkets and malls |  |
| **Convenience** | proportion consuming street food due to convenience | Fish knowledge and convenience, Physical environmental influences | Distance to the nearest supermarket; possible impacts on the diets of the catchment community in Kisumu; type of market outlet; type of retail outlets where the food was purchased; number of days in a week that street foods and kiosk foods were consumed; consumption of non-home foods; market distance; distance to town center with supermarket purchase | Time to prepare healthy foods, online food purchase; Time to prepare traditional food for working parents; availability of microwaves instead of regular gas ovens (makes processed foods more acceptable) | Presence of home delivery; Time preparing food; Ease of food preparation | Convenience influences dietary pattern, Cooking at home perceived as time consuming; Using sustainable foods requires more effort to acquire which is not convenient | Cooking methods among adults; Proximity of vegetable stands, shops or malls to purchase vegetables to residence; Reasons for changes in food consumption practices - taste of food (fresh vs packaged, processed or frozen food) |  |
| **Desirability** | Food preferences; taste acceptability; sensory appeal of food | Individual (intrapersonal),social environmental (interpersonal), physical environmental (community settings), and macrosystem (societal) open interview questions, Beliefs about fish; Willingness to eat, Attitudes of conventional consumers, Quinoa beliefs, Preferences for six characteristics: price, salad, soup, sides (rice and beans), meat and fruit | Preferences for milk and milk products; who people eat with and where (synchronisation); Drivers and barriers of food consumption; something that makes healthy eating difficult for you; (iii) something that makes healthy eating easy for you; (iv) something that influences what you eat in your (local) area; and (v) a person that influences what you eat in your (local) area; porridge flour choice; consumer choice; “something/situation that makes eating healthily difficult for you”; iii. “something/situation that makes eating healthily easy for you”; iv. “something/situation that influences what you eat in your area”; and v. “a person that influences your food or drink choices in your area”; 8-10 pictures and/or photographs that represent your thoughts and feelings about the safety of vegetables; preferences for traits such as color and nutritional quality; diversity of leafy AIVs at retail outlets, | Attitudes toward halal food purchase, subjective norms (social pressure to comply with halal food purchase expectations), perceived behavioral control (being in control over making halal food purchases), self-efficacy; attitude towards organic foods, subjective norms (social norms around organic food purchase), perceived social support (parents, relatives, or friends supportive of purchasing organic food); Shifting cultural preferences around eating out, peer influence on food preferences; Perceived knowledge of liquid milk risk, attitudes towards purchasing liquid milk; freshness of food; Taste; Knowledge of food safety (16 questions); Color, smell of foods; Knowledge about eating practices; Customer satisfaction with restaurant food; Reasons customers buy food from street food vendors; Attitudes towards organic foods; social norms, attitudes; Preferences toward lower vegetable intake, preferences toward junk food & soft drinks among university students; facilitators of fast food preference; popularity of celebrating special occasions at fast food restaurants | Attitude to milk quality from retail outlets; Preparation/consumption options for raw milk; Attitudes towards milk fat content; Familiarity with milk production system; Usage of milk for other purpose; Child food preferences; Cultural factors affecting consumption; Mother's nutrition and food safety knowledge; Mother's religious background; Food choice; Nutritional value; Food safety; Health; Taste; Familiarity; Food taboos; Preferences for raw meat and milk ; Knowledge of risk of consumption of raw meat and milk; Food safety beliefs; Types of cereals preferred by urban consumers | Preferences determine food choice. Children like “taste of the food” and “the attractive appearance of the food at first sight”; People desire certain qualities in food items aligned with their values such as being environmentally conscious; Fruit and vegetable consumption trends as proxy for preferences | Food preferences; Influence of other people on child daily food intake; self-reported taste preferences; Student perceptions of fast food - how often they eat it, what they think of it in relation to harming their heath; Reasons for changes in food consumption practices - preference for meat dishes and the frequent consumption of packaged goods; Changes in preference for fresh and healthy food |  |
